# Philosophical producers, philosophical consumers, and the metaphilosophical value of original texts

**DOI:** 10.1007/s11098-022-01900-8

**Published:** 2022-10-29

**Authors:** Ethan Landes

**Affiliations:** Philosophy Department, University of Zurich, Zürich, Switzerland

**Keywords:** Intuitions, Thought experiments, Method of cases, Textual analysis, Testimony, Metaphilosophy

## Abstract

In recent years, two competing methodological frameworks have developed in the study of the epistemology of philosophy. The traditional camp, led by experimental philosophy and its allies, has made inferences about the epistemology of philosophy based on the reactions, or intuitions, people have to works of philosophy. In contrast, multiple authors have followed the lead of Deutsch and Cappelen by setting aside experimental data in favor of inferences based on careful examination of the text of notable works of philosophy. In other words, the debate is split between authors focusing on philosophy’s *consumption* and those focusing on philosophy’s *production*. This paper examines the motivation for focusing on original texts and other evidence of philosophy’s production and finds it lacking. Drawing upon Hills’ distinction between propagation and transmission, I argue that the social epistemology of philosophy does not justify the recent focus on original texts of philosophy. Because the philosophical knowledge of consumers of philosophy is likely *inspired* by producers of philosophy, as opposed to epistemically grounded in the producers’ epistemic states, experimental philosophy had it right all along—if we want to know the epistemic standing of philosophy, we need to look to philosophy’s consumers.

## Introduction

Looking at how analytic philosophy has been carried out over the last few decades, there is no question that analytic philosophers use thought experiments to try to convince other philosophers of their theses. It turns out, however, that this is about the only uncontroversial thing that can be said about philosophers’ use of thought experiments.

Among both epistemologists of philosophy and analytic philosophers in general, the received view of the epistemology of thought experiments holds that thought experiments provide justification and knowledge of philosophical claims by eliciting intuitions relevant to the truth of those claims. While there has proven to be considerable disagreement about the nature of intuitions and how exactly intuitions underpin our beliefs, most philosophers working on the topic have taken philosophers’ widespread use of language like "it is intuitive that P" or “my intuition is that P” as reflecting the underlying epistemic reality of thought experiments.

This received view about intuitions’ role in philosophy is not without its critics (Cappelen, 2012; Deutsch, 2015; Horvath, 2022; Ichikawa & Jarvis, 2013; Williamson, 2007). In particular, Cappelen (2012, 2014a, 2014b) and Deutsch (2009, 2010, 2015, 2016), and recently Horvath (2022) have attracted considerable attention for arguing that the role of intuitions in philosophy has been overstated because not enough care has been paid to what the texts containing original presentations of thought experiments actually say. Indeed, when we look at the original presentations of notable thought experiments like Gettier (1963), Foot (1967), or Thomson (1976), not only is there little to no mention of intuitions, but extended discussions of the cases typically precede and follow the actual thought experiments. These extended passages are where Deutsch, Cappelen, and Horvath contend that justification about philosophical theses actually resides. Authors of thought experiments do not rely on intuitions to justify their claims, Deutsch, Cappelen, and Horvath argue. Instead, authors of thought experiments rely on arguments. Because of this, Deutsch, Cappelen, and Horvath contend that the traditional focus on intuitions in explanations of the epistemology of philosophy has been misplaced.

This line of argument, while having gained significant attention, has proven unpopular among other metaphilosophers (see Horvath, 2022 for a recent overview). To pick just a few lines of criticism, text-based denial of intuitions has been criticized for misidentifying the sorts of mental states intuitions are (Weinberg, 2014), for failing to explain the historical details of epistemology after Gettier (Brown, 2017), for failing to appreciate that stated evidence does not equate actual evidence (Egler, 2020), and for entailing skepticism about philosophy’s ability to evaluate texts (Landes, 2020). However, while much has been said criticizing the conclusion that intuitions do not play a role in philosophy, the methodological choice to argue from wording of original texts has not been examined.

Explaining the choice to examine original texts, Deutsch says,

The important methodological question is: What methods are employed by *good* philosophers, ones who, by fairly wide consensus, have made interesting and important philosophical progress, increasing, in a significant way, our body of philosophical knowledge? (Deutsch, 2015, p. 41)

In other words, Deutsch argues that there is something particularly important about paying attention to original and groundbreaking works of philosophy. The original texts are more metaphilosophically relevant than anything “downstream”, such as how these texts are understood or how they are discussed by other philosophers.

Both Cappelen and Horvath adopt similar positions, albeit less explicitly. For example, in criticizing the negative experimental philosophy of Swain et al. (2008), which experimentally studied intuitions about Lehrer’s (1990) Mr. Truetemp cases, Cappelen says,

Swain et al. make two false assumptions about Lehrer’s paper: [First,] *Lehrer’s* judgment that Mr. Truetemp does not know is based on an intuition. [Second,] the failure to account for that intuition *is used* as an argument against reliabilism. [emphasis added] (Cappelen, 2012, pp. 222–223)

Cappelen is arguing from the epistemic status and methods of Lehrer himself, as presented in Lehrer’s, 1990 book. According to Cappelen, Swain et al.’s discovery of order effects in judgements about Mr. Truetemp cases does not matter to the debate about reliabilism because *Lehrer* does not rely on such judgements as evidence.

This paper argues that the above focus on original texts is misguided. The social epistemology of philosophy is such that there can be a large gap between the justification presented in a work of philosophy and the justification a reader gains from reading the work of philosophy. This is because a work of philosophy can cause a belief in a reader without being the epistemic basis of that reader’s belief. Works of philosophy instead generally inspire readers to evaluate the claims made in the works by the readers’ own lights. In the language of Hills (2020), philosophical texts can *propagate* knowledge in readers without *transmitting* knowledge to readers. Therefore, attempts to uncover the methods or justification of a work’s author or the methods or justification presented in a work’s text reveal little of value about the epistemic standing of philosophy more generally.

In Sect. 2, I introduce the view underlying the quotes above and discuss its importance in contemporary debates on the epistmology of philosophy. In Sect. 3, I examine possible routes to defend producer-focused metaphilosophy, namely a passage in Deutsch (2015) that appeals to progress. Then, after rejecting Deutsch’s argument, I offer an alternative defense based on testimony. In order to better understand testimony’s role in philosophy, Sect. 4 introduces Hills’ (2020) distinction of propagation versus transmission and applies it to philosophical communication. Section 5 argues that when we look at how philosophers learn from each other, testimony is unnecessary and possibly fairly rare. Section 6 ties everything together, arguing that examining original texts reveals little of interest to current metaphilosophical debates.

## Producer-focused metaphilosophy

In arguing against the role of intuitions in philosophy, Deutsch, Cappelen, and Horvath take what is said in original texts as providing key insights into the epistemology of philosophy. Moreover, all three acknowledge the popularity of the belief—both among metaphilosophers and analytic philosophers at large—that intuitions are key to justifying our verdicts about thought experiments. Deutsch, Cappelen, and Horvath nonetheless take text-based evidence as decisive, or nearly so, against the role of intuitions. In this move, there is a key methodological innovation in what sorts of evidence matters to debates about the epistemology of philosophy. By focusing on original texts as a way to gain insight into in the epistemic standing of philosophy, Deutsch, Cappelen, and Horvath are giving metaphilosophical priority to the facts about how philosophy is *produced*—facts about the author and the author’s writing. Call this view *producer-focused metaphilosophy*.

Producer-focused metaphilosophy holds that original works of philosophy carry metaphilosophical importance that consumption or later discussion of the text does not. Specifically for the purposes of the epistemology of philosophy, potential defeaters—such as those provided by experimental philosophy—are irrelevant unless they defeat justification that actually exists in the original texts. Producer-focused metaphilosophy can be contrasted with *consumer-focused metaphilosophy*, which places prime metaphilosophical importance on the consumption (i.e., reading or listening) of works of philosophy. Experimental philosophy, traditionally understood, is a type of consumer-focused metaphilosophy, as it studies and draws inferences from the judgments people have in response to reading texts or other philosophical stimuli.^1^

Producer-focused metaphilosophy is essential for text-based denial of intuition’s role in philosophy. Suppose that original presentations of thought experiments do present non-intuitive evidence for the verdicts of thought experiments. It is a further claim that evaluation of this non-intuitive evidence is important for evaluating the methods of philosophy because it may be that the actual contents of a text are nearly or completely epiphenomenal to later practice. The conclusions of many influential analytic texts are often discussed and taught without actually being read (much to the collective frustration of Deutsch, Cappelen, and Horvath). People who have never read *Naming and Necessity*, for example, believe that Kripke disproved descriptivist theories of reference using a series of thought experiments involving mistaken definite descriptions, and they believe it because this is how the main thrust of Book 2 of *Naming and Necessity* is presented in classrooms and at the pub. These later presentations and discussions of texts may be the source of knowledge and justification for most or all philosophers.^2^ Producer-focused metaphilosophy thereby screens off the relevance of these later presentations and consumption of the material, thus justifying the move Deutsch, Cappelen, and Horvath make from text-based evidence to a claim about philosophical practice more generally.

While producer-focused metaphilosophy is required to motivate exegesis-based rejections of intuition-based philosophy, it has avoided critique. In fact, producer-focused metaphilosophy has been adopted by many critics of text-based intuition denial (e.g., Brown, 2017; Chalmers, 2014; Chudnoff, 2017; Colaço & Machery, 2017; Landes, 2020). In one particularly illustrative example, Chalmers (2014) adopts a producer-focused stance toward his own work on philosophical zombies to argue against Cappelen’s anti-intuition reading of Chalmers’ work:

In that presentation, I first appeal to the conceivability of zombies, saying I take this to be intuitively obvious (and therefore noninferentially dialectically justified), but I go on to give a number of arguments for this claim from underlying principles (for example, the nonanalyzability of consciousness in functional terms). (Chalmers, 2014, p. 540)

Because Chalmers is the producer of the work being analyzed, Chalmers is trying to beat Cappelen at Cappelen’s own producer-focused game. While Cappelen analyzes the methods of Chalmers (1996) (the text) through exegesis, only Chalmers (the producer) has first-hand knowledge of the methods of Chalmers (1996) (the text). Using this first-hand knowledge, Chalmers takes *his own methods* to ultimately defend the use of intuitions in philosophy. As the rest of this paper will argue, however, Chalmers should have instead looked to his readers for answers.

## Defending producer-focused metaphilosophy

While producer-focused metaphilosophy has been adopted by philosophers working on the literature on intuitions, it has to my knowledge only been explicitly defended at any length by Deutsch (2015). This section looks at Deutsch’s defense of producer-focused metaphilosophy, rejects it as relying on questionable and problematic assumptions about academic progress, then offers a defense in its place based on testimony that will be the focus of the rest of the paper.

Deutsch is aware that the distinction between producers and consumers is important to his rejection of intuition’s role of philosophy, relying on the distinction in a key defense of his method of examining original texts (Deutsch, 2015, pp. 98–99). Moreover, Deutsch anticipates the distinction drawn above between producer-focused and consumer-focused metaphilosophy, and offers what is in essence a defense of producer-focused metaphilosophy:

The core methods of the discipline, and of any discipline, are reflected most clearly by the most clearly successful examples of discovery and progress in the discipline. A focus on such examples in philosophy is entirely appropriate. (Deutsch, 2015, p. 41)

Unfortunately for producer-focused metaphilosophy, this claim about methods and progress is probably not true. The methods of disciplines are not reliably reflected in the most notable examples of progress because progress has often occurred when core methods were tweaked or set aside. Darwin’s *On the Origin of Species* (1859/2011) employs an argument from analogy between domesticated animals and wild animals even though arguments from analogy are rarely used in contemporary biology. Turning to thought experiments, thought experiments are fairly uncommon in contemporary primary scientific literature. Nonetheless, some of the greatest advances in physics—whether the shift to Newtonian mechanics, the adoption of special and general relativity, or the introduction of quantum mechanics—involved thought experiments (Gendler, 2000; Kuhn, 1977). Similarly, there are many norms of philosophy *not* reflected in Gettier’s, 1963 rejection of the JTB account of knowledge. Most obviously, few other published philosophy papers have successfully defended a thesis in 1000 words.

Setting aside this worry about whether extraordinary works of a discipline use ordinary methods, the rate at which philosophers misunderstand each other raises a second objection to this sort of defense of producer-focused metaphilosophy. Philosophers misread, mishear, and misunderstand works of philosophy all the time. Even some of the best-studied works of philosophy are either subject to intractable disagreements about textual interpretation or undergo major textual reinterpretations. For an example of the former, Hume scholars have long disagreed about the fundamental goal of Hume’s discussion of causation and the extent to which Hume is making metaphysical, epistemic, or psychological claims about causation (see Russell, 2008, pp. 3–11). For an example of the latter, Plato’s use of myth in his dialogues was seen for decades to be a vestigial leftover of older ways of thinking (Buxton, 1999) but has recently undergone reinterpretation as a key aspect of his arguments (Most, 2012; Murray, 1999). These are just two examples of texts that have proven both influential in the history of philosophy and (assuming there is only one correct interpretation) subject to widespread misunderstanding. Given such phenomena, producer-focused metaphilosophers must then explain how even influential and widely-examined texts like those of Hume and Plato can be so broadly misunderstood yet remain more metaphilosophically relevant than the epistemic states of the people reading them.

Third, and related to the second objection, if intuition deniers are right and philosophers have mistakenly thought that intuitions are used as evidence in philosophy, then philosophers have been widely mistaken about the methods of clearly successful examples of progress. This leaves Deutsch, Cappelen, and Horvath in the awkward position of accepting that progress has occurred but philosophers have been widely mistaken about what the progress is grounded in or e﻿ven consists of. Some philosophical accounts of progress might allow for this,^3^ but even if we accept that progress can occur in philosophy when a substantial majority of philosophers have false beliefs about what the progress consists of, we still have reasons to not take original texts very seriously. Assume that authors of notable thought experiments did not rely on intuitions but successfully discovered philosophical truths. Assume further that consumers reading these texts thought that intuitions were involved and treated their intuitions as putative warrant for the conclusions being defended. Then metaphilosophers still have to accept that many philosophers’ beliefs have been caused by unreliable methods, leading them to defend false beliefs and to explore blind alleys (see Nado, 2016).

Given the problems with Deutsch’s argument about progress, what a defense of producer-focused metaphilosophy needs is a strong epistemic connection between producer and consumer. Without it, the position faces the problems just discussed, where the methods and epistemic status of original texts come apart from the methods and epistemic status of those consuming the texts. While analysis of original texts would thereby tell us interesting historical facts about how producers presented their own epistemic states, it would not tell us anything about the epistemic standing of philosophers writ large.

Testimony, at least of the right kind, would provide exactly the necessarily strong epistemic connection between producers and consumers needed for producer-focused metaphilosophers to justify their focus on original texts. In particular, producer-focused metaphilosophers need *transmission* of epistemic states from producer to consumer, which is a strong account of testimony often found among anti-reductionists about testimonial knowledge (Coady, 1992; see Greco, 2016; Hills, 2020). Transmission accounts of testimony take the speaker’s own knowledge to be what epistemically grounds the listener’s knowledge, as opposed to, say, the listener’s personal judgments about the speaker’s reliability. If transmission occurs between philosophical producers and philosophical consumers, examining original texts is a great tool for determining our epistemic states. In this case, our epistemic states about the thesis defended in a work of philosophy are grounded in the epistemic states of the work’s producer, and so examining original texts is the best evidence we have of what the producer—and by extension we—know.

It is worth pausing to head off concerns that any account that relies on the existence of philosophical testimony is a non-starter. Speaking anecdotally, philosophers are often wary of the idea that they gain philosophical knowledge through testimony. In the same that way testimonial knowledge of an artwork’s beauty seems problematic (see Hopkins, 2011; Robson, 2012), there is a sense that philosophers’ reliance on testimony would constitute a professional failing or a failure of intellectual virtue. Nonetheless, there are two main reasons to think that testimony plays a key role in our consumption of other people’s philosophy.

First, a substantial amount of our knowledge in other domains depends on the knowledge of other people. I have never been to Spain, but my knowledge that Barcelona is sunny in the summer and cloudy in the winter depends (at least in large part) on what my Spanish and Catalan friends have told me. Closer to philosophy, logicians and mathematicians seem to have testimonial knowledge of a priori and necessary facts based on the work of one another. Unless we have reason to think that philosophy is different from other domains, denying philosophical testimony risks entailing the implausible claim that testimonial knowledge in areas related to philosophy, such as mathematics and logic, is impossible (Ranalli, 2020).

Second, philosophers *act* as if we gain philosophical knowledge through testimony. While work in philosophy is piecemeal, it is nonetheless interrelated. Advances in one topic affect advances in another, and our philosophical reasoning employs assumptions based on works of other philosophers that we lack relevant expertise, time, or desire to evaluate. The most charitable reading of this behavior is that we are taking our beliefs as justified by the testimony of experts in other sub-disciplines. Which advances we treat as if we know through testimony will differ from philosopher to philosopher, but potential examples include that possible worlds are the right way to handle modal reasoning and modal semantics, that the law of non-contradiction holds, or that ZFC set theory is most likely self-consistent. If it turns out that we do not know these propositions through testimony despite our use of them in our philosophizing, we have a devastating result for the epistemic standing of philosophers.^4^

## Transmission and propagation

In this section, I continue discussion of testimony in philosophy by introducing a key distinction drawn by Hills (2020) between learning from another person through *transmission* and learning from another person through *propagation*. Paying attention to the different avenues knowledge can be spread from person to person is particularly important in a metaphilosophical context because the social epistemology of statements in philosophy differs from the social epistemology of statements in many other areas (Anscombe, 1979; Moran, 2006, pp. 279–280; Wanderer, 2013). In particular, philosophers communicate to each other through arguments that often do not rely on specific empirical claims about the world. This is important for understanding how philosophical consumers learn from the works of philosophical producers.

To illustrate the social epistemology of philosophical arguments, consider this toy example of a Moorean anti-skeptical argument:

I know that I have a hand. My hand is external to my mind, so if I know I have a hand, I know there is an object external to myself. Given closure of known entailments, if I know there is an object external to myself, I know there is an external world. Therefore, I know that there is an external world.

The toy argument does not map nicely onto the examples philosophers usually give of testimony. Compare the toy argument to examples from the testimony literature, such as being told it is cold outside (Moran, 2006, p. 278), reading first-hand accounts of religious miracles (Hume, 1748), receiving directions to the post office (Coady, 1992, p. 38), and a self-proclaimed clairvoyant telling a friend that Elvis is alive in San Diego (Lackey, 2008, p. 16). In these cases, we might have reasons to trust or distrust what is asserted, but this trust or distrust falls far short of our ability to evaluate the premises and conclusion of the toy argument. This is because claims in the toy argument are evaluable to the consumer in a way the canonical examples of testimony are not. The sorts of propositions communicated in traditional cases of testimony are propositions that we as listeners must go out of our way to check for ourselves—if they are even within our epistemic grasp at all. In contrast, when reading the anti-skeptical argument above, we are able to evaluate the claims being defended on the fly. We can evaluate for ourselves whether the premises entail that we know there is an external world, whether the argument begs the question, whether closure of known entailment holds, and so on.

The distinction between the toy argument and the cases from the testimony literature is not as clear-cut as it might first appear, however. How much they resemble each other depends on how the details of the cases of testimony are filled in. Consider Coady’s example of asking directions to the post office (1992, p. 38). I may not know the town at all and just urgently need to buy stamps while on vacation. Because I do not know the town, when I get directions, I have no choice but to rely on the speaker. As far as I know how to find the post office, I know because of the speaker’s testimony. Imagine instead that I have been in town a few days and want directions to double-check the route I think is best. In this second case, my epistemic standing is much closer to my epistemic standing when reading the Moorean argument. I may not have perfect knowledge of the town, but I might still know enough from my time there to evaluate whether or not the person I asked for directions is confused or lying to me. Nonetheless, if the directions I receive match up with what I already believe about the town, the speaker's testimony can still add justification for my belief about how to best reach the post office.^5^

Philosophical arguments and some instances of testimony (such as the latter case of directions to the post office) are evaluable by the consumer because there are two separate epistemic pathways through which communication can cause knowledge. In related discussions of moral testimony, Hills (2020) distinguishes between *transmission* and *propagation*. Transmission corresponds to how testimonial knowledge has been discussed thus far in the paper; a consumer learns a proposition by transmission if it is epistemically grounded on the producer’s knowledge (Hills, 2020, p. 401). In contrast, when knowledge is propagated, a consumer gains knowledge by employing non-testimonial methods that in turn ground their knowledge (Hills, 2020, p. 401). The distinction between transmission and propagation is found in all forms of communication and corresponds to the everyday distinction of showing versus telling (Grice, 1957). For example, if we were speaking and I wanted you to know what was in my pockets, there are two epistemically distinct ways I could cause you to know that proposition. First, I could tell you (i.e., transmit the proposition) that I have my keys and my phone in my pocket. In contrast, I could also pull the keys and phone out of my pocket and show you (i.e., propagate the proposition).

The most important aspect of the distinction for present purposes is that transmission and propagation result in knowledge with different epistemic bases. In instances of transmission, a consumer’s knowledge is based on a producer’s knowledge that p, whereas in cases of propagation, the consumer’s knowledge is merely caused by the producer’s assertions. Instead, when propagation causes knowledge, the knowledge is epistemically based on whatever non-testimonial evidence a consumer themself brings to bear to evaluate what the producer says. Returning to the example of causing you to know I have keys in my pocket, in the former case of transmission, your knowledge about the contents of my pocket is epistemically grounded in my testimony, whereas in the latter case of propagation, your knowledge is epistemically grounded in your own perception. Similarly, if philosophical producers transmit knowledge to consumers, the consumers’ knowledge is based on the knowledge of the producers, whereas if philosophical producers propagate knowledge, consumers’ knowledge is based on the consumers’ own evaluation of the claims being made by the producer. What exactly the basis of philosophical consumers’ knowledge is in cases of philosophical propagation is itself contentious, but it will be whatever the ultimate grounds of philosophical knowledge are, whether intuitions, reasons, arguments, inferences, a combination of these, or something else entirely.

Despite the epistemic and conceptual differences between transmission and propagation, in practice they are not mutually exclusive, and they can even interact. For example, when I tell you I have keys in my front pocket, you may be assured of my testimony based on looking for and seeing the outline of a keychain.^6^ Moreover, propagation can provide defeaters for testified propositions. For example, if I lie to you and the lie inspires you to check for the truth of my claim by your own lights, knowledge caused by propagation provides a defeater for my testimony. When propagation defeats transmission, we have source-sensitive defeaters (Casullo, 2003; Constantin & Grundmann, 2020) that remove reasons to believe the transmitted proposition on the basis of the producer’s testimony. This source-sensitive defeat does not defeat the evidence itself, but it defeats the connection between the evidence (that the producer says such and such) and the proposition (the information testified). This does not defeat our own independent evidence for believing a proposition, but rather defeats our justification for the proposition based on our evidence that *the testifier said such and such*.

## Philosophical progress without testimony

With the key distinction between transmission and propagation in place, it is time to turn specifically to the social epistemology of works of philosophy. To have the sort of strong epistemic connection between producers and consumers needed to defend producer-focused metaphilosophy in the way discussed in Sect. 3, transmission does not need to be the only way knowledge spreads between philosophers, but it needs to be the primary way knowledge spreads from original works of philosophy. If the spread of knowledge from original texts is dominated by propagation, then the basis of the philosophical knowledge gained from interacting with texts will depend primarily on the consumer. In this case, examining original texts for signs of the methods and epistemic standing of the producer will at best tell us little more than that—the methods and epistemic standing of the producer.

In this section, I argue that transmission is unnecessary to explain how philosophers learn from each other. Propagation is sufficient to explain how consumers gain knowledge from producers, and there are initial reasons to think that propagation is, as a matter of fact, the primary way philosophers communicate. To start, consider the following thought experiment:

In the early 1960s, a man decides to write a prank academic paper. The man chooses words from then-contemporary epistemology papers, including “justified”, “the”, “knowledge”, “know”, and “Jones”. He writes these words on pieces of paper, sets the pieces of paper on the floor, and puts pieces of fish on each paper. He then sets his cat down in the middle of the floor, letting the cat wander from piece of fish to piece of fish. As the cat eats a piece of fish, the man writes down the corresponding words, in order, on a piece of paper. Once the cat has had its fill of fish, to the man’s delight, the resulting string of words looks uncannily like actual epistemology. With this in mind, and keeping the order of words untouched, the man formats the result, adds punctuation, and, with a snicker, submits the paper to *Analysis* under the name of his cat, Edmund Gettier.

The editor at *Analysis* sends the paper off to a referee and receives a report back stating that the argument is pithy, succinct, and apparently sound. The referee even reports that the paper has convinced her that JTB is not sufficient for knowledge, despite her previous belief to the contrary.

The man who submitted the paper to *Analysis* comes clean and admits what he did. The editor passes this information on to the referee, who responds to the editor by saying “So what? I now know that JTB is not sufficient for knowledge.”

Despite having any putative transmission defeated by the author’s insincerity, the referee nonetheless seems to know the proposition defended in the paper, even upon learning of the hoax.^7^ To see why, consider the general argumentative structure of Gettier (1963). First, a view—the JTB account of knowledge—is introduced. Then, two counterexamples are presented. Finally, the paper infers from the counterexamples that JTB is not sufficient for knowledge. Every step of this argument is something the consumer can evaluate by their own lights. Therefore, because the referee does not need testimony to know any proposition asserted in the paper, as long as she considers the argument herself and does not rely on the words of the author, her knowledge is maintained despite her knowledge being caused by the text of the hoax.

How exactly the referee considers the argument by her own lights comes down to the question of what justifies our knowledge when we think through arguments containing thought experiments. This is, of course, the very issue at the heart of the literature being discussed in this paper. Intuition-based accounts of the epistemology of thought experiments will hold that a key step of the text is available for the consumer (in this case, the referee) to evaluate because thought experiments elicit intuitions in the consumer. On such accounts, the consumer can treat the communication as propagation because intuitions provide the consumer with independent access to justification about whether the character in Gettier (1963) has justified true belief that is not knowledge. This is not to say that the verdict of the thought experiment is the only part of Gettier (1963) (or any other work of philosophy) that is consumer-evaluable and thus able to be propagated. On an intuition-based account of the epistemology of philosophy, it is also within the consumer’s epistemic abilities to evaluate for themselves whether, for example, the intuited verdict is a genuine counterexample to the JTB account of knowledge, whether the overall argument begs the question, or whether a step is missing in the overall argument.

Whether or not the consumer can evaluate the argument by their own lights does not depend on intuitions being a key epistemic resource in philosophy. In their positive discussions of the epistemology of philosophy, Deutsch, Cappelen, and Horvath still grant that the sorts of philosophical texts considered here are accessible from the armchair and thus evaluable by consumers’ own lights. In terms of consumer-evaluability, Deutsch, Cappelen, and Horvath merely differ from intuition-based accounts in that they place more emphasis on texts’ arguments than texts’ thought experiments. Therefore, most of what I have said above about the consumer-evaluability of the Moorean argument and the larger Gettier argument applies to intuition-free accounts of philosophy as well.

To illustrate, consider Cappelen’s (2012, pp. 139–148) reading of Burge's (1979) arthritis case, in particular Burge’s claim that a patient in our social environment who says "I have arthritis in my thigh*"* has the same conceptual content corresponding to “arthritis” as we do, despite their mistaken beliefs about arthritis. Cappelen traces Burge’s justification for this claim to the *empirical fact* that language users will make mental state attributions like “the patient thinks they have arthritis in their thigh” (Cappelen, 2012, p. 144). Even though the argument’s justification is empirical, it still allows for propagation. What matters to whether an act propagates knowledge is whether the consumer already has the necessary evidence *or* collects the evidence necessary to evaluate the claim by their own lights. Many empirical claims are beyond our ability to evaluate by our own lights, but analytic philosophers generally try to provide arguments that we as consumers can evaluate for ourselves. Accordingly, even though Cappelen takes Burge’s argument as resting on an empirical observation, consumers, as mature language users, are in the same epistemic position as Burge to evaluate whether that empirical observation is correct.

My argument that philosophers can learn from each other by propagation admittedly falls short of demonstrating that propagation is the only pathway for philosophical communication. First, empirical claims that are beyond the consumer’s ability to judge for themselves do work their way into philosophical arguments. Second, propagation and transmission are not mutually exclusive, so the possibility of propagation does not rule out that transmission ever occurs in philosophical settings. Therefore, what has been said so far is consistent with the claim that, when it comes to thought experiments and other philosophical arguments, philosophical consumers gain knowledge from producers via both propagation and transmission.

Nonetheless, the epistemic environment of philosophy means we should be skeptical that the necessary conditions for transmission are in place, especially for the sorts of texts producer-focused metaphilosophers have been focusing on. Because of contingent features of the field of philosophy, consumers often have a reasonable expectation of peer disagreement, defeating testimony (Ranalli, 2020; Sliwa, 2012).^8^ This does not defeat philosophical testimony across the board, but does suggest that in many instances testimonial knowledge is impossible or at least epistemically irresponsible (Ranalli, 2020). Indeed, the works of philosophy that philosophers hold as the most important are usually those that changed people’s minds. Because of this, the sorts of original works that producer-focused metaphilosophers have been scrutinizing are works that defend theses that the producers’ epistemic peers disagree with, at least at the time of publication. In other words, consumers’ epistemic states upon reading a work of philosophy, especially if it is not one of the few works of philosophy widely accepted to be sound, are akin to the referee’s upon reading the cat-generated philosophy paper.^9^

## Metaphilosophers should not focus on original texts

As discussed at the start of the paper, producer-focused metaphilosophy arose as a method to argue that intuitions do not play a central role in philosophy. Despite producer-focused metaphilosophy’s partisan origin, producer-focused metaphilosophy has since been adopted by metaphilosophers on both sides of the debate about the centrality of intuitions in contemporary analytic philosophy. With my arguments in place, we can now answer whether or not the use of producer-focused metaphilosophy is justified in this debate in the first place. That is, should philosophers interested in how philosophers gain knowledge take producer-focused approaches by examining what is written in the original presentations of thought experiments and other texts?

The sufficiency of propagation in philosophical communication means that the answer is no—the actual wording of original presentations of thought experiments does not tell us much at all about the epistemology of philosophy. When reading works of philosophy, we are not beholden to testimony in the way we might be when reading about scientific findings or works of history. Thought experiments and the arguments surrounding them are something we as consumers can evaluate for ourselves using our own non-testimonial epistemic tools. When learning this way, by propagation, we do not so much gain our justification from what texts *actually* say as much as we gain justification from what we *think* the texts are asking us to consider. Therefore, original texts can ultimately serve as *inspiration* for the beliefs of consumers without being the *epistemic basis* of those beliefs. If we are perfectly capable of forming knowledge based on our own epistemic tools and without testimony, then the actual epistemic status of an author or original text may in fact be epiphenomenal to everyone who reads that text.

Due to significant potential for a gap between a text’s stated justification and the justification inspired by the text, determining the epistemic states caused by a text requires a consumer-focused approach rather than a producer-focused approach to the epistemology of philosophy. The epistemic grounds of consumers' beliefs contingently rely on what they considered while reading a text. Therefore, if we want to know whether or not philosophy is on solid epistemic footing, we need to study what sorts of epistemic processes consumers use when reading works of philosophy. Perhaps they generally base their beliefs on certain cognitive states, perhaps they usually carefully consider arguments, or perhaps philosophers generally just take a producer's’ word for what is stated and move on. We cannot know until we study the consumption of philosophy.

Even if philosophers do generally gain knowledge from texts via transmission, we need to take a consumer-focused approach to determine that this is the case. Determining if texts transmit knowledge requires examining consumers’ psychology and epistemic environment. Transmission cannot occur in what Hills calls “an atmosphere of doubt” (2020, p. 406). For Hills, an **“**atmosphere of doubt” is a normative notion. Reason to doubt, whether or not we appreciate that we have reason to doubt, defeats testimonial knowledge. Thus, if we consume philosophy in an atmosphere of doubt—such as one full of peer disagreement or skeptical worries from experimental philosophy—we cannot gain philosophical knowledge via transmission. Importantly, whether there is an atmosphere of doubt can change over time. Peer disagreement waxes and wanes, and epistemically problematic experimental findings are made or fail to replicate. Therefore, whether or not transmission is defeated requires examining the *epistemic environment of the consumer* to determine whether such defeaters give the consumer reason to doubt.

In addition, doubt descriptively prevents transmission. If a consumer thinks they should doubt a producer, they will approach a work with a far more critical eye. Instead of taking the producer’s word for it, a doubting consumer will instead employ their own epistemic tools to evaluate the producer’s claims. Consumers’ doubt— justified or unjustified—will limit transmission. Therefore, even if philosophers know the claims of notable thought experiments via transmission, we cannot know this until we look at the attitude consumers take toward the text. Moreover, in a field like analytic philosophy, where students are taught to approach texts critically, we should expect to find high levels of doubt among consumers.

It is worth pausing here to clarify the scope of my claims. I am not denying that careful textual analysis can still be epistemically valuable. I am denying that careful textual analysis is valuable in the way producer-focused metaphilosophers think it is. Even if we only learn from philosophers via propagation, careful textual analysis can lead to richer propagated philosophical knowledge, since textual analysis can improve what we consider by our own lights. Similarly, reading secondary literature that draws our attention to subtleties in a primary text can help our own efforts at learning via propagation by highlighting things we might not have otherwise considered. We can even use textual analysis to learn about authorial intent—as Deutsch, Cappelen, and Horvath do. While there are reasons to be skeptical that authors accurately convey their own methods in their written work (see Brown, 2017, p. 196; Egler, 2020, pp. 3361–3362; and footnote 7, above), work by historians of philosophy demonstrate that sophisticated analysis of authorial intent is possible. Nonetheless, when we are careful about the social epistemology of philosophy and the role propagation may play in spreading knowledge from producer to consumer, we can see that authorial intent does not tell us much at all about the epistemic states of consumers.

Propagation is not just a problem for producer-focused metaphilosophy as a metaphilosophical method—it also challenges the anti-intuition position that producer-focused metaphilosophy was originally developed to defend. While Deutsch, Cappelen, and Horvath are right that seminal presentations of thought experiments do not usually make reference to intuitions and contain passages that can be interpreted as arguments for particular verdicts, finding arguments in said texts does not tell us if those arguments have a downstream effect on consumers’ epistemic states. Perhaps the arguments are ignored, misunderstood, or fail to provide justification. Indeed, some initial consumer-focused experimental work on the issue suggests that arguments do not play a large role in consumers' epistemic states around texts involving thought experiments. Wysocki (2017) examined whether or not arguments following thought experiments cause people to change their verdicts about the thought experiments and found the arguments following the case had no significant effect on participant verdicts (see, however, Horvath (2022) for methodological concerns about the study). Given the role of propagation in the consumption of philosophy, these findings suggest that it does not matter if producers point to non-intuitive evidence in written arguments that follow presentations of thought experiments—that is not where consumers’ justification is coming from.

Here we can return to an issue I raised in Sect. 3 against Deutsch’s defense of producer-focused metaphilosophy and explain with greater fidelity why it is a problem that accounts that reject intuitions’ justificatory role in philosophy run against the self-conception of the last couple decades of analytic philosophy. Consider what Horvath says when contemplating why rejections of intuitions’ central role in philosophy have proven unpopular among metaphilosophers:

The most charitable explanation that I can come up with is that analytic (meta)philosophers are still so much in the grip of the intuition-based view of the method of cases that they tend to automatically reinterpret Gettier (1963) and other seminal texts in this light. (Horvath, 2022, p. 9)

If philosophical consumers learn from philosophical producers via propagation, then this (mis)conception of analytic philosophy may well have been self-fulfilling. Analytic philosophers have read texts *thinking* they ought to base their belief on intuitions. This has likely lead philosophers to focus their attention on thought experiments and pay less attention to the passages around thought experiments. It has also likely lead philosophers to lend extra weight to their verdicts about thought experiments over other considerations presented in said works. Therefore, to the extent that belief revision is directly and indirectly within our control, philosophers’ belief that intuitions are crucial evidence in philosophical theorizing has led to intuitions playing a crucial role in belief formation in analytic philosophers.

## Conclusion

Recently, many metaphilosophers have begun paying close attention to the exact wording and methods of original presentations of notable philosophical thought experiments. Deutsch, Cappelen, and Horvath have in particular argued that the absence of intuition-talk in these texts lends evidence to the conclusion that intuitions do not play a key justificatory role in philosophy. This paper argues that the use of original texts to argue for broad claims about the epistemology of philosophy is on shaky ground. We should understand philosophers as gaining inspiration from original texts as opposed to gaining knowledge directly transmitted from texts or their authors. Philosophers’ justification therefore often rests on what epistemic resources they as consumers bring to bear when considering whether or not a philosophical argument is sound. Therefore, without examining *how* people consumed a work of philosophy, examination of original texts may merely provide us with evidence about the epistemology of the texts’ authors instead of revealing something more fundamental about the epistemology of philosophy.

## Footnotes

1 Experimental philosophy is only contingently consumer-focused, as experiments that study the psychology behind the creation of thought experiments could be devised.

2 When talking about “philosophical knowledge”, “philosophical justification”, and “philosophical testimony”, I specifically mean knowledge and testimony about the target of philosophical inquiry. These are substantive, object-level claims about the sorts of things philosophers investigate as philosophers. Compare this with claims about philosophy as a discipline. “Berkeley’s idealism is false” is an example of the sort of proposition I am interested in investigating the epistemology of, while “Berkeley’s idealism is widely considered to be a major development of British empiricism” – a claim about the field of philosophy – is not. I am assuming for present purposes that we can have philosophical knowledge.

3 Bird (2007) may be one such account. Appealing to Deutsch’s use of “progress” in the passage quoted above is one possible route for defending producer-focused metaphilosophy that I leave unexplored here. It does, however, face two immediate hurdles. First, evaluative metaphilosophy has traditionally focused on warrant, justification, or knowledge of philosophical propositions instead of other epistemic states such as progress or acceptance. To defend producer-focused metaphilosophy this way, we need reason to shift the focus of evaluative metaphilosophy to these other epistemic states. Second, such a defence of producer-focused metaphilosophy still needs some way of screening off later misunderstandings as metaphilosophically unimportant. Progress is likely not much good to a community of inquirers if most or all of the inquirers misunderstand the progress, such as mistakenly thinking that intuitions are involved in discovery.

4 One might object that when we make assumptions based on the work of others, we are drawing conditional inferences from which base claims seem best or most likely to be true. Because we often take up theoretical assumptions based on the work of others, this would mean a large chunk of philosophical knowledge is conditional as opposed to non-conditional, but some philosophers—especially those with Quinean and/or anti-realist leanings—might find this palatable. Such a move removes the need to justify the antecedents of conditionals via testimony, but it does not completely remove the need for testimony in philosophy. Even if philosophical knowledge is mostly conditional, we still often lack the relevant expertise, time, or desire to determine what is entailed by antecedents.

5 Notice that there is a risk of double-counting evidence when I and the testifier both have the same evidence for p and I nonetheless take their testimony as additional evidence for p. Thanks to Jan Constantin for this point.

6 Following Lackey (2008), I am using “testimony” in a way that is neutral on the epistemology of the claim. This allows me to talk about speech acts that purport to transmit knowledge without committing to whether the acts successfully transmit.

7 Notice I am not explicitly referencing intuitions here to support the verdict. This is not because I do not think intuitions are playing a role in convincing you the reader that the referee can maintain her knowledge here. I *do* think reactive judgements about the case will convince many readers that the referee maintains knowledge. I am avoiding intuition-talk for pragmatic reasons. To name a few such reasons, I am avoiding intuition-talk in part because I think intuition-talk is clunky, in part because I do not want readers to think my argument here relies on a partisan account of intuitions, and in part because some readers may draw upon non-intuitive methods here, which I do not want to discourage.

8 For empirical work on the extent of disagreement, see the PhilPapers surveys on philosophical beliefs (Bourget & Chalmers, 2014, 2022). Note there is disagreement about how optimistic or pessimistic to be about the results (e.g., Grundmann, 2013; Ranalli, 2020). For example, Ranalli is optimistic about the results, pointing out that experts generally agree on a lot of topics, such as whether anti-skepticism is true. It is a mistake to read too much into this agreement, however. Surface-level agreement often hides underlying disagreement, and anti-skeptics are anti-skeptics for a wide range of incompatible reasons.

9 The discussion of peer disagreement and testimony in philosophy deserves much more space than can be afforded here. Readers worried that my point about peer disagreement is self-defeating should see (Everett, 2014; Henderson et al., 2017; Kornblith, 2013; van Inwagen, 2010) for a sampling of the different ways around the challenge of peer disagreement in philosophy.
